# Omnidirectional color wavelength tuning of stretchable chiral liquid crystal elastomers

**DOI:** 10.1038/s41377-024-01470-w

**Published:** 2024-05-22

**Authors:** Seungmin Nam, Wontae Jung, Jun Hyuk Shin, Su Seok Choi

**Affiliations:** Department of Electrical Engineering, Pohang University of Science and Technology (POSTECH), Pohang, Korea

**Keywords:** Liquid crystals, Displays

## Abstract

Wavelength-tunable structural colors using stimuli-responsive materials, such as chiral liquid crystals (CLCs), have attracted increasing attention owing to their high functionality in various tunable photonic applications. Ideally, on-demand omnidirectional wavelength control is highly desirable from the perspective of wavelength-tuning freedom. However, despite numerous previous research efforts on tunable CLC structural colors, only mono-directional wavelength tuning toward shorter wavelengths has been employed in most studies to date. In this study, we report the ideally desired omnidirectional wavelength control toward longer and shorter wavelengths with significantly improved tunability over a broadband wavelength range. By using areal expanding and contractive strain control of dielectric elastomer actuators (DEAs) with chiral liquid crystal elastomers (CLCEs), simultaneous and omnidirectional structural color-tuning control was achieved. This breakthrough in omnidirectional wavelength control enhances the achievable tuning freedom and versatility, making it applicable to a broad range of high-functional photonic applications.

## Abstract (web summary)

On-demand color control, enabling simultaneous and omni-directional wavelength tuning towards both shorter (blue-shift) and longer (red-shift) wavelengths, is reported using stretchable chiral liquid crystal elastomers.

## Graphical abstract

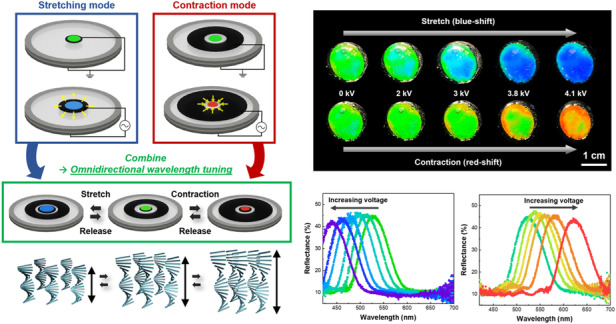

## Introduction

Structural colors by the photonic band gap^1–3^ in nano-periodic dielectric structures and the wavelength tunability of structural colors using stimulus-responsive materials have attracted increasing research interest for various tunable photonic device applications^4–8^. Especially, chiral liquid crystals (CLCs), which can be obtained from their self-organized helicoidal nanostructures^9–12^, offer large-scale high-quality structural colors bypassing conventional nano-fabrication. Moreover, according to the De Vries equation^10^, CLCs enable precise and wide-ranging wavelength tuning by modifying the helical pitch length via external stimuli^13–15^. This capability, combined with high optical quality and easy fabrication process, establishes CLCs as a promising material for tunable structural colors in photonic applications^16–20^. However, it is important to note that most CLC wavelength-tuning studies have focused solely on unidirectional wavelength control rather than omnidirectional wavelength tuning^21–32^. The most experimental reports to date have approached a helix winding wavelength-tuning mechanism toward shorter wavelengths^24–26^. This is because the helix winding of CLCs with pitch contraction is more uniform in large sizes, and various triggering methods are possible, such as thermal^27,28^, optical^29,30^, and electrical stimulation^31,32^.

A flexible and stretchable chiral liquid crystal elastomer (CLCE) can be synthesized through the configuration of a CLC within an elastomeric framework by employing reactive mesogenic molecules^33,34^. The structural color of the CLCE can be effectively modulated in the broadband wavelength range through direct application of mechanical stretching deformation, leading to large helix distortions. Diverse approaches have been adopted to utilize the broadband wavelength tunability and stretchability of CLCEs since the pioneering work on tunable CLCEs reported by Finkelmann et al.^35^. These studies have led to the development of advanced applications for stretchable CLCEs, such as photonic camouflage^36^, chameleon-like e-skin^37,38^, biomedical sensors^39,40^, and elastomeric fiber technologies^41,42^. Moreover, the control of CLCEs in matrix driving of multi-pixel array operations has been reported recently^43^. However, most of the previous reports on wavelength tuning using stretchable CLCEs have been limited to a single directional wavelength shift toward shorter wavelengths rather than multidirectional tuning, which would allow for both broadband and completely unrestricted wavelength control. Based on the theoretical framework proposed by Warner and Terentjev^44,45^ and recent experimental investigations of the optical properties of CLCEs under multi-axial stretching deformation^46^, it is hypothesized that the stretching deformation of CLCEs induces pitch-contractive helix reconfiguration, resulting in a reduction in the optical period. Despite active research on CLCE color tuning through mechanical deformation, there has been a definite limit to the unidirectional blue shift toward shorter wavelengths through pitch contraction^47–51^.

In light of the ideal wavelength tuning of structural colors, omnidirectional wavelength shifts toward not only shorter wavelengths but also longer wavelengths direction rather than only toward shorter wavelengths is highly preferred to maximize the tuning range and on-demand degree of freedom of the wavelength tunability. Moreover, considering that storing free energy is radically increased against helix contraction, using only pitch-tightening helix control is assumed to be an obstacle to maximizing the wavelength tuning range. Therefore, the critical technical hurdle in realizing omnidirectional wavelength tuning for CLCs or CLCEs lies in the systematic achievement of uniform helix unwinding, which leads to the wavelength of the structural color lengthens because of the elongation of the pitch while preserving the contractive properties of the pitch. As mentioned previously, the helix unwinding theories suggested by de Gennes and Kawachi^21,22^ are limited experimentally because of the non-uniformity and vulnerability of the CLC structure under an in-plane field. In contrast, the singular action of stretching the standing helix within the CLCE initiates helical pitch contraction, leading only to a blue shift in the structural color toward shorter wavelengths.

Herein, we report the experimental realization of omnidirectional structural color tuning toward longer and shorter wavelengths with significantly improved broadband wavelength control using a stretchable CLCE. In contrast to the conventional approach involving areal stretching with a positive strain perpendicular to the CLCE helix and subsequent pitch contraction, the contractive areal deformation in this study induces a negative strain, facilitating pitch-expanding deformation of the CLCE. Hence, an intuitive conceptualization is established concerning the structural color shift toward longer wavelengths with pitch expansion. To achieve realistic areal contractive deformation with negative strain in the CLCE, a novel methodology was employed. Specifically, the CLCE was positioned on an electro-active dielectric elastomer actuator (DEA) featuring compliant electrodes arranged in a surrounding donut-shaped configuration. This allowed for a red shift of the structural color changes toward longer wavelengths through electrical controllability. Moreover, by placing a circular-shaped electrode on top of the compliant circular-shaped electrode of the DEA to operate the same CLCE, a heterogeneous configuration could be established. In this configuration, a positive strain could also be induced in the standing helix, resulting in a pitch-contractive blue shift of the structural color toward shorter wavelengths. Thereby, simultaneous and bi-directional wavelength control involving a red shift toward longer wavelengths and a blue shift toward shorter wavelengths of the structural color could be achieved concurrently. Moreover, the ideally desired omnidirectional structural color change in a single CLCE device was successfully realized by employing on-demand expanding and contractive DEAs featuring stacked donut-shaped and circular plane-compliant electrodes.

## Results

### Positive and negative strain of the CLCE for omnidirectional wavelength tuning

Most studies on wavelength-tunable structural colors using soft actuators have focused on reducing the optical period through areal stretching actuator deformation, which results in only positive strain of the stretchable structural colors. In CLCE, this stretchable positive strain leads to pitch contraction and following wavelength shift toward shorter wavelengths, as explained by Warner and Terentjev^44,45^. Nevertheless, to extend the optical period and secure a longer wavelength shift in stretchable CLCEs, the application of a negative strain for areal contractive deformation is imperative. This requirement is considered to be a pivotal technological challenge for realizing omnidirectional wavelength tuning.

**Fig. 1:**
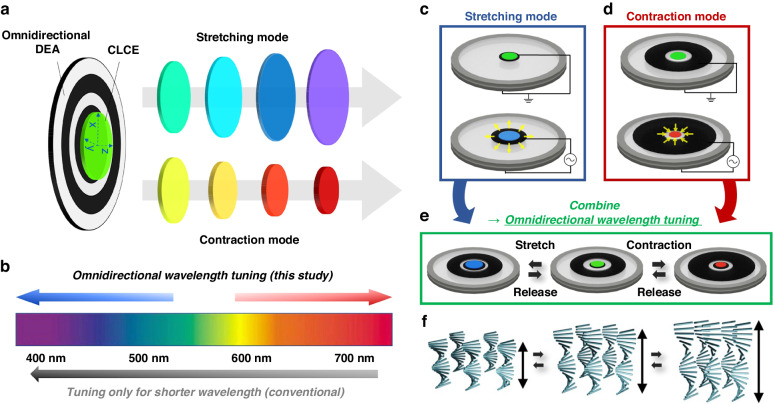
Mechanism of omnidirecitonal wavelength tuning using an electoactive stretchable CLCE. **a** Schematic representation of the omnidirectional color wavelength-tunable device, consisting of a mechanochromic CLCE and electrically deformable DEA. **b** Reflection wavelength tuning toward longer and shorter -wavelengths. c-f) Schematic representations showing operations of the DEA in (**c**) contraction mode, (**d**) stretching mode, and (**e**) omnidirectional mode by combining the contraction and stretching modes; (**f**) helix deformation of the CLCE under each tuning method

The operational principle of the omnidirectional color wavelength-tunable CLCE device is illustrated in Fig. 1. To realize both positive and negative strains of the CLCE, we developed a multi-mode operating stretchable soft actuator that generates not only expanding deformation with positive strain, but also contractive deformation with negative strain of the stretchable CLCE to achieve omnidirectional wavelength control. Electrically controlled omnidirectional structural color tuning was successfully realized by combining a multi-mode actuating DEA that is capable of radially stretching the central expansion deformation and central contraction in a donut-shaped actuating deformation (Fig. 1a, b).

When the DEA operates in the radial stretching mode to generate a positive strain in the CLCE, the area in the x/y-plane of the CLCE should expand, and its length in the thickness direction (z-direction) should decrease owing to volume conservation. This reduction in thickness causes the helical pitch length of the CLCE to contract proportionally, resulting in a wavelength shift toward shorter wavelengths (blue-shift), as illustrated in Fig. 1c. Conversely, in the contraction mode with donut-shaped stretching actuation, when the DEA generates a negative strain in the CLCE, the area of the CLCE decreases, while its thickness increases. Thus, the helical pitch length of the CLCE should also increase, and a shift toward longer wavelengths (red-shift) of the CLCE can be obtained (Fig. 1d). Omnidirectional wavelength tuning of the CLCE can be realized by employing a multi-mode actuating DEA that can produce two types of stretching and contractive deformations (Fig. 1e). To summarize, the omnidirectional control of pitch contraction for shorter-wavelength tuning and pitch expansion for longer-wavelength tuning was investigated using a single CLCE and a multi-mode actuating DEA (Fig. 1f).

### Bi-modal stretching deformations of the DEA for positive and negative strain effects

**Fig. 2:**
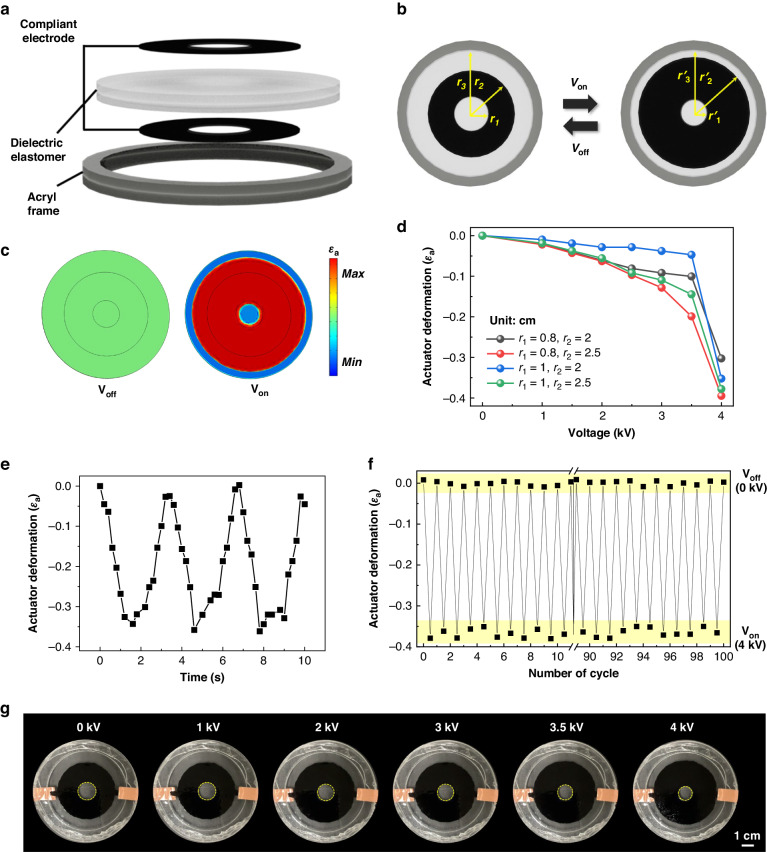
Electro-mechanical deformation under contraction-mode DEA operation. **a** Schematic representation of the vertical structure of the DEA for negative strain. Donut-shaped compliant electrodes are utilized for contractive deformation. **b** Top view of the contraction-mode DEA before and after voltage application (*r*_1_ > *r*ʹ_1_, *r*_2_ < *r*ʹ_2_, *r*_3_ = *r*ʹ_3_). **c** FEA simulation results for the contraction-mode DEA with an applied voltage of 4 kV. **d** Contractive deformation of the DEA (*ε*_a_) as a function of the applied voltage when the geometric structure of the compliant electrode (*r*_1_, *r*_2_) is varied. **e** Time-dependent contractile deformation of the DEA (*ε*_a_) with the geometric structure fixed at the optimal condition (*r*_1_ = 0.8 cm, *r*_2_ = 2.5 cm). **f** Repeatable driving under an applied voltage of 4 kV for up to 100 cycles. **g** Macroscopic images at various applied voltages

Conventional DEAs are traditionally employed solely to induce substantial stretching deformations characterized by a positive areal strain^52,53^. However, to facilitate longer-wavelength tuning of the CLCE, it is necessary to develop a DEA capable of areal contractive deformation, thus enabling the application of negative strain to the CLCE while increasing the pitch length. A conventional DEA produces stretching deformation by expanding the area of the compliant electrodes due to Maxwell stress across the dielectric elastomer when an electric field is applied^53^. Notably, areal-expanding deformations with a positive strain occur only in the actuating area with compliant electrodes. In contrast, the adjacent passive area without compliant electrodes undergoes areal contractive deformation owing to the pushing strain effect when the electroactive DEA stretching is initiated. Thus, the generation of contractive deformation, which induces negative strain in the CLCE, can be achieved by exploiting the passive region of the dielectric elastomer where stretching deformation is absent. This passive region serves as the primary utilization area for implementing contractive deformation (Fig. 2). Additionally, to achieve uniform and symmetrical areal contractive deformation, a donut-shaped compliant electrode structure on the DEA was designed by utilizing the area where compliant electrodes were not placed (Fig. 2a). Upon application of a voltage exceeding the threshold, the active stretching area of the donut-shaped compliant electrode expands symmetrically in the in-plane direction, similar to a conventional DEA. Notably, the area of the central circle is reduced because the donut-ring actuation extended both inward and outward. Consequently, the active area with the compliant electrode increases from *π*(*r*_2_^2^ - *r*_1_^2^) to *π*(*r*ʹ_2_^2^ - *r*ʹ_1_^2^), and the adjacent passive area of the center circle with no electrode should contractively decrease from *πr*_1_^2^ to *πr*ʹ_1_^2^ (Fig. 2b). Therefore, the experimental validation successfully demonstrated the attainment of a negative strain via the contraction-mode DEA. The contraction-mode DEA and resulting negative-strain effect were also verified using finite element analysis (FEA) methods (Fig. 2c and S1). The strain distribution results from the FEA under an applied voltage provide a clearer understanding that contraction deformation was induced at the center as the donut-shaped compliant electrode area was actuated (Video S1). It is crucial to consider that the effective deformation of the DEA may vary depending on the size and shape of the compliant electrode and rigid frame combination^54^. Therefore, we systematically investigated the effect of actuator deformation while varying the radii of the electrodes, *r*_1_ and *r*_2_ (Fig. 2d). Factor *r*_3_, representing the diameter of the acrylic rigid frame, remained fixed at *r*_3_ = 3.5 cm. It was found that the smaller the inner diameter, *r*_1_, and larger the outer diameter, *r*_2_, of the donut-ring electrodes, the greater the contraction deformation effect in the center position of DEA was obtainable. The optimal conditions were set as *r*_1_ = 0.8 cm and *r*_2_ = 2.5 cm, where the maximum areal contraction with a negative strain of -0.4 was observed.

To examine the stability and repeatability of the contraction-mode DEA over the time scale, we meticulously observed the effective actuating deformation of DEA (*ε*_a_) as a function of time under application of a 4 kV sine wave electrical signal at 0.3 Hz to the optimized DEA device (Fig. 2e). The results demonstrate stable, repetitive, and symmetric driving operations without any hysteresis. Furthermore, we validated the area change rate and macroscopic images by performing contraction–relaxation repeated actuations for up to 100 cycles to confirm the practical applicability of the contraction-mode DEA (Fig. 2f and S2). The area of the circle at the center position of the donut-shaped electrodes should shrink as a function of the applied voltage, resulting in negative strain and contractive deformation (Fig. 2g and Video S2). Although the actuation strain of the contraction-mode DEA was slightly lower than that of the conventional stretching-mode DEA^55^, it was sufficient to induce continuous and stable device operation with a negative strain for longer-wavelength tuning.

### Tuning of the structural color toward longer wavelengths using a stretchable CLCE

**Fig. 3:**
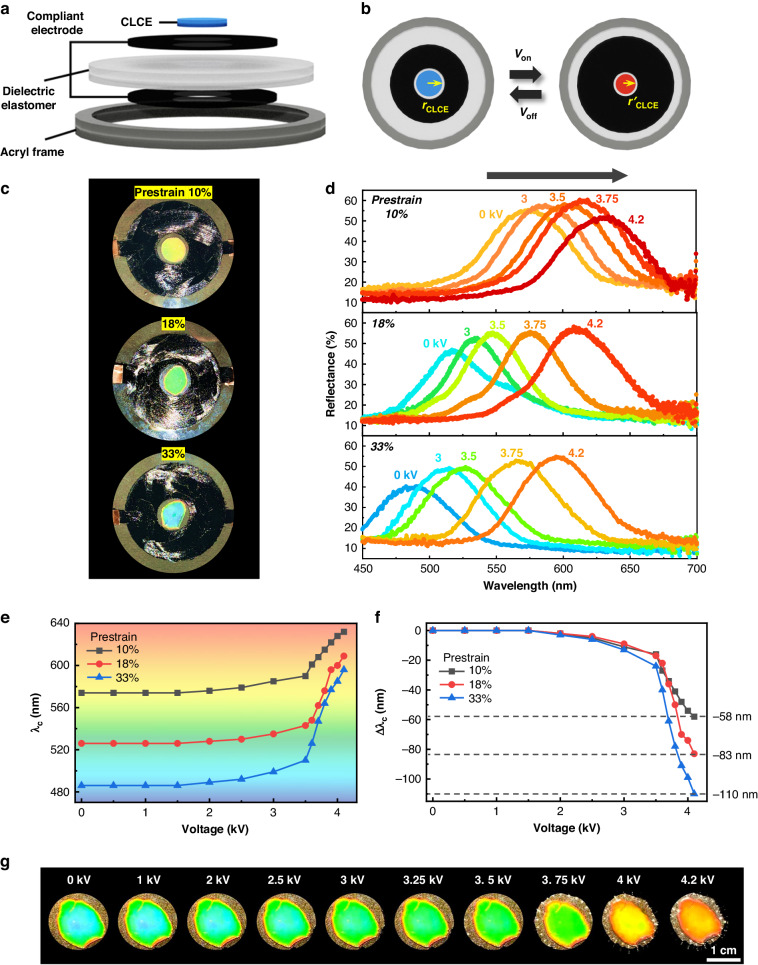
Electro-optical response of the contraction-mode CLCE. **a** Schematic representation of the vertical structure of the contraction-mode CLCE. **b** Top view of the contraction-mode CLCE before and after voltage application (*r*_CLCE_ > *r*ʹ_CLCE_). **c** Macroscopic images showing the color of the CLCE with pre-strains of 10%, 18%, and 33%. **d** Reflection wavelength shift toward longer wavelengths according to the applied voltage. **e**, **f** Central wavelength (**e**) and central wavelength shift (**f**) as a function of the applied voltage. **g** Structural color changes at various applied voltages

To achieve control of longer-wavelength shifts utilizing the negative-strain effect, a pre-stretched stretchable CLCE was fabricated as a hybrid device with the optimized contraction-mode DEA illustrated in Fig. 2 (Fig. 3a). The well-known thiol-acrylate synthesis method^56,57^ was employed to prepare the stretchable CLCE (Fig. S3). It should be noted that a sufficient amount of pre-stretching of CLCE is imperative. Without pre-stretching, the maximum wavelength shift caused by contractile deformation cannot be achieved because of the problematic out-of-plane buckling effect, which disrupts the continuous pitch-widening helix deformation of the CLCE (Fig. S4). By applying an electrical voltage to this hybrid device composed of a pre-stretched CLCE on a contraction-mode DEA surrounded by donut-shaped compliant electrodes, red-shifted structural color tuning of the CLCE toward longer wavelengths can be achieved. During this longer-wavelength tuning of the CLCE, the area of the pre-stretched CLCE should decrease from *πr*_CLCE_^2^ to *πr*ʹ_CLCE_^2^, while the thickness increases to satisfy volume conservation (Fig. 3b). Therefore, the helical pitch length also increases in proportion to the thickness expansion, resulting in an experimentally implemented longer-wavelength shift.

However, the pre-strain magnitude of the CLCE plays a pivotal role in determining the specific values of the initial central wavelength and the associated wavelength shift range. To elucidate the practical implications of the pre-strain in the CLCE under contraction-mode actuation, we systematically examined the electro-optical behaviors that influence wavelength changes. This comprehensive analysis covered areal pre-strain conditions of 10%, 18%, and 33%. Under further pre-stretching conditions, the initial reflection color exhibited an enhanced blue shift, corresponding to the expected decrease in the pitch length (Fig. 3c). To determine the wavelength, change characteristics when the contraction-mode DEA was operated, the reflection spectra and central wavelength (*λ*_c_) were observed as functions of the applied voltage for each CLCE pre-strain condition (Fig. 3d, e). The central wavelengths in the initial state (*λ*_i_) and the maximum contraction state (*λ*_m_) according to the pre-strain of the CLCE (*ε*_pre_) were (*ε*_pre_ = 10%; *λ*_i_ = 574 nm, *λ*_m_ = 632 nm), (*ε*_pre_ = 18%, *λ*_i_ = 526 nm, *λ*_m_ = 609 nm), and (*ε*_pre_ = 33%, *λ*_i_ = 486 nm, *λ*_m_ = 596 nm). In brief, the experimental results confirm that an increased pre-strain applied to the CLCE corresponds to greater wavelength tunability, as evidenced by a larger Δ*λ*_c_ value (Fig. 3f). The broadband tuning potential of the CLCE for longer wavelengths was evident, reaching a maximum Δ*λ*_c_ of 110 nm at 4.1 kV in the *ε*_pre_ = 33% pre-strain configuration. The longer wavelength tuning of the stretchable CLCE was apparent through a shift from the initial blue-green color to an orange color (Fig. 3g and Video S3).

### Continuous and repeatable longer-wavelength tuning of the contracting CLCE

In pursuit of ideal omnidirectional wavelength control, the need for both continuous and repeatable wavelength shifting is essential, encompassing both shorter- and longer-wavelength tuning processes. However, considering the helix deformations of CLCE, concerns arise regarding the continuous, repeatable, and stable nature of the pitch-widening process during longer-wavelength tuning without irreversible breaking of the helix structure. It is noteworthy that previous reports have confirmed the continuity and repeatability of the pitch contraction process during shorter-wavelength tuning^58,59^.

**Fig. 4:**
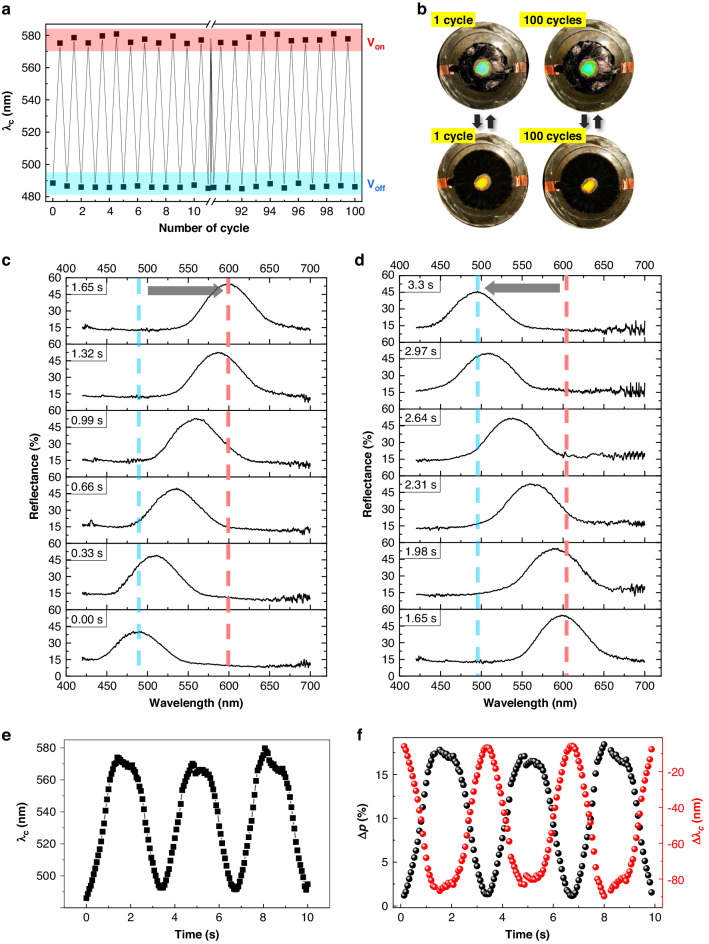
Time-dependent monitoring of the electro-optical response of the contraction-mode CLCE. **a** Repeatable wavelength tuning under an applied voltage of 4 kV for 100 cycles. **b** Photographs of the contraction-mode CLCE after 1 cycle and 100 cycles. **c**, **d** Time-dependent reflection spectrum at the voltage with 0.3 Hz sine wave 4.1 kV. **c** contractile and (**d**) relaxation state. **e**, **f** Central wavelength position, (**e**) central wavelength shift and pitch variation (**f**) during a continuously applied voltage of 4 kV in a 0.3 Hz sine wave for up to 10 s

To address these concerns, we carefully monitored the time-dependent wavelength-shifting process during longer-wavelength tuning of the stretchable CLCE, as shown in Fig. 4. At the maximum longer-wavelength condition, corresponding to a 33% pre-strain and 4 kV voltage with 0.3 Hz actuations, the repeatability of switching was confirmed over 100 cycles of the longer-wavelength tuning process (Fig. 4a). The initial central wavelength of *λ*_c_ ≈ 490 nm and the longer shifted central wavelength of about *λ*_c_ ≈ 580 nm at the maximum contraction condition were maintained during these repeated switching cycles. The visual representations of the switching device at cycles 1 and 100 are identical, as shown in Fig. 4b. No discernible hysteresis was observed in the switching of structural color.

Furthermore, for an in-depth exploration of the precise evolutionary changes in the longer-wavelength shifting process, we investigated the reflection wavelength spectra during both the contraction and recovery switching phases of the CLCE. This analysis was conducted for the entire switching duration by applying a 4 kV electric voltage in a 0.3 Hz sine wave conditions (Fig. 4c, d). The results conclusively demonstrate a continuous red-shift switching process toward longer wavelengths throughout the entire deformation time of the CLCE from 0 s to 1.65 s. Additionally, the recovery process to transition back to the original wavelength position occurs from 1.65–3.3 s upon removal of the electric voltage.

Both the On and Off switching processes of this longer-wavelength control were symmetrical and reversible without the loss of chiral reflection properties. Moreover, it was confirmed that the reflection central wavelength, *λ*_c_, wavelength shift, Δ*λ*_c_, and estimated pitch change, Δ*p*, during three driving cycles were fully continuous, symmetrical, and reversibly stable (Fig. 4e, f). As confirmed by the reflection spectra and wavelength shift, continuous and stable wavelength tuning was possible in the electrically tunable contraction-mode CLCE. Therefore, it is believed that this longer-wavelength control can be manipulated in a manner similar to the previously confirmed shorter-wavelength tuning method to achieve omnidirectional wavelength control.

### Simultaneous wavelength tuning device with opposite wavelength shifts

**Fig. 5:**
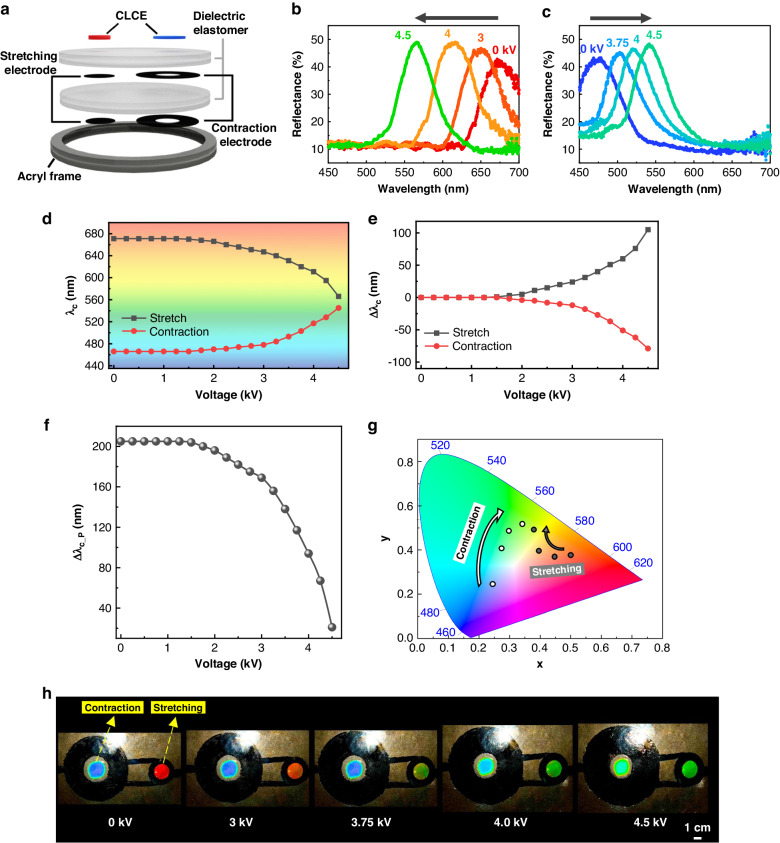
Electro-optical response of the hybrid-mode CLCE. **a** Schematic representation of the vertical structure of the hybrid-mode CLCE. b,c) Reflection wavelength shift toward (**b**) longer wavelengths of the contraction pixel and (**c**) shorter wavelengths of the stretching pixel. **d**, **e** Central wavelength (**d**) and central wavelength shift (**e**) as a function of the applied voltage. **f** Central wavelength difference between the contraction and stretching pixels. **g** Reflection color change in the CIE 1931 color space under an applied voltage. **h** Vivid color contrast control of the opposite-wavelength shifting hybrid CLCE as a function of the voltage

Based on the results for the longer-wavelength control of the CLCE, single-device integration was investigated to allow simultaneous longer- and shorter-wavelength control. Through hybridization of the independently functioning contraction-mode CLCE illustrated in Fig. 3 and the stretching-mode CLCE detailed in our previous study^58^, we successfully developed a single device capable of simultaneous tuning for both longer- and shorter-wavelength control, as shown in Fig. 5. It is important to note that in the hybrid single device, the same DEA is utilized throughout the switching process (Fig. 5a). Upon applying electric voltages of up to 4.5 kV at 0.3 Hz in a sine wave, simultaneous opposite wavelength changes were clearly observed (Fig. 5b, c). The central wavelength of the stretching pixel was adjusted from its initial value of 671 nm to 566 nm, achieving a maximum wavelength tuning magnitude (Δ*λ*_c_) of 115 nm. The contraction pixel was tunable from the initial 466 nm to 545 nm, representing a tuning range of 79 nm in the same time (Fig. 5d, e). As noted previously, the tuning power of the stretching mode was slightly superior to that of the contraction mode. However, simultaneous and opposing wavelength changes within this hybrid device configuration were observed to manifest in an almost identical manner.

Meanwhile, the contrast in the structural color in terms of the central wavelength difference (Δ*λ*_c__p) between stretching-pixel CLCE and contraction-pixel CLCE can be adjusted as a function of the applied voltage (Fig. 5f). In the Off state, a considerable color contrast, marked by a 205 nm wavelength difference, was reduced to a 26 nm difference in the On state during operation at 4.5 kV. This implies that color difference can be controlled from a vivid distinguishable state to an indistinguishable state. For additional quantitative color comparison, the color contrast was estimated using the color coordinate calculation for each CLCE pixel in the CIE 1931 color space (Fig. 5g). By modulating the applied voltage, the initial vivid color contrast between red and blue was systematically altered, converging toward a similar green color (Fig. 5h and Video S4). Therefore, color pixel control using this hybrid approach can potentially be utilized for photonic applications such as color pixel binning, pixelated camouflage, or multiple wavelength sensor applications following this study.

### Realization of on-demand omnidirectional wavelength tuning

**Fig. 6:**
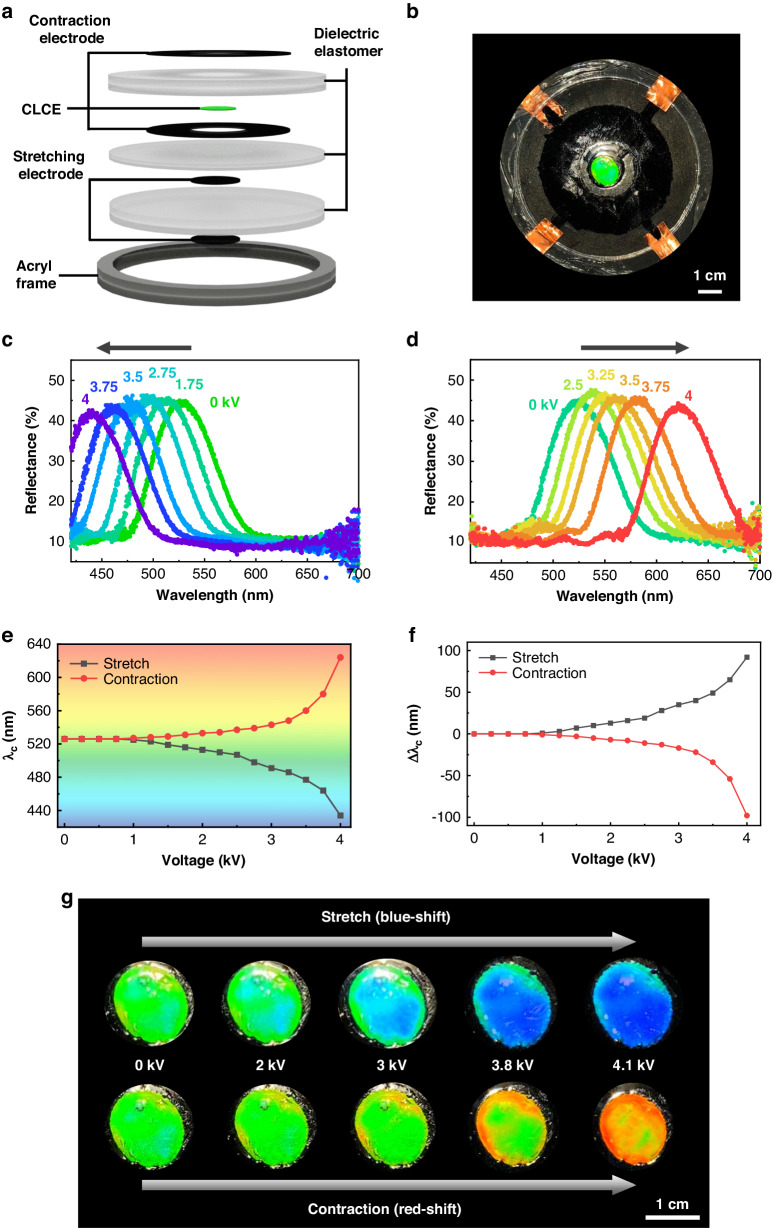
Electro-optical response of the omnidirectional-mode CLCE. **a** Schematic representation of the vertical structure of the omnidirectional-mode CLCE. **b** Top view of the omnidirectional-mode CLCE before voltage application. **c**, **d** Reflection wavelength shift toward (**c**) shorter wavelengths using the stretching mode and (**d**) longer wavelengths using the contraction mode. **e**, **f** Central wavelength (**e**) and central wavelength shift (**f**) as a function of the applied voltage. **g** Omnidirectional structural color changes at various applied voltages

Beyond simultaneously controlling the opposite wavelength tuning mechanisms, we devised an omnidirectional wavelength-tunable CLCE device to achieve on-demand ideal wavelength control and maximize tunability freedom, as illustrated in Fig. 6. To build a singular device functioning in an identical position, we stacked the stretching- and contraction-mode DEAs into a single device (Fig. 6a). To prepare the omnidirectional-mode CLCE device, we utilized a pre-stretched CLCE with an initial medium wavelength set to green. This configuration allowed for investigation of the bi-directional wavelength tunability, including shifts toward both longer and shorter wavelengths within the visible wavelength range (Fig. 6b). Upon application of an electric voltage, clear shifts in the reflection spectra were observed in the same single CLCE, with distinct shifts toward both shorter and longer wavelengths (Figs. 6c, d, S5, and Video S5). The electro-active stretching mode of DEA induced a positive strain, tuning the CLCE toward shorter wavelengths. Conversely, electrically actuating the contraction mode of the DEA induced a negative strain, tuning the same single CLCE toward longer wavelengths.

Remarkably, the wavelength shifting behavior in both opposite directions appeared to be symmetrical in this omnidirectionally tunable device mode, as observed through the reflection central wavelength (*λ*_c_) and the corresponding shift magnitude (Δ*λ*_c_) (Fig. 6e, f). The initial central wavelength of the single CLCE at *λ*_c_ = 526 nm was bi-directionally tuned to 624 nm for the contraction mode and to 434 nm for the stretching mode (Fig. 6f). Regarding the wavelength shift magnitude (Δ*λ*_c_), both the contraction mode and stretching mode exhibited similar tuning ranges of 98 nm and 92 nm, respectively. Thus, utilization of the omnidirectional tuning method was verified to yield a notably expanded wavelength tuning range compared with the approach employing only unidirectional wavelength tuning. Unlike the majority of studies focused on improving the tuning power of stretchable structural color in CLCEs through synthetic material development, our approach to omnidirectional wavelength control aims to achieve a maximized tuning range, almost doubling the performance. Furthermore, it is important to note that a single stretching tuning method requires significantly stronger tuning power or the development of a much less elastic material. This is essential for addressing the continuous increase in the tuning resistance to the deformation energy in a stretching-only CLCE tuning system. In contrast, the omnidirectional tuning system proposed in this study enables a significantly reduced requirement for tuning power because it addresses the chiral deformation in both pitch tightening and pitch expansion. Therefore, during stretching mode operation, we achieved a tunable color change from the initial green to deep blue; in contraction mode, a distinct color shift from the initial green to orange was observed (Fig. 6g and Video S6). It is anticipated that further potential tuning can be achieved across the entire and possibly beyond the visible wavelength range through optimization of the synthetic materials of the DEA and CLCE, following this report of omnidirectional wavelength control.

## Discussion

Omnidirectional wavelength control represents an advanced solution for overcoming technological restrictions in structural color-based optical devices. By using the structural color of a CLCE, we investigated the simultaneous and omnidirectional control of color wavelengths in both the longer- and shorter-wavelength directions. To realize the opposite directional control of omnidirectional wavelength tuning, both positive and negative strain-inducing methods were devised by controlling the effective deformation of the CLCE helix on an electroactive DEA. In addition to the positive strain applied to the CLCE by the conventional stretching-mode DEA with circular-shaped compliant electrodes, a negative strain on the CLCE was achieved by the novel contraction-mode DEA with donut-shaped ring expanding compliant electrodes developed using the same materials. As a result, a longer-wavelength shift of the CLCE due to the negative strain effect with a uniform shorter-wavelength shift of the CLCE due to the positive strain was obtained using bi-modal operation of the DEA. The opposite bidirectional wavelength shift of the CLCE was reversible, repeatable, and symmetrical in each direction. The bidirectional wavelength shifts were also confirmed through the simultaneous pixelated hybrid CLCE structures with opposite directional wavelength shifts in the same time. Furthermore, when the hybrid DEA structure of the stretching and contraction operation modes was constructed with a vertical stacking configuration, an independent and on-demand omnidirectional wavelength-tunable CLCE could be obtained. The proposed omnidirectional-mode CLCE demonstrated stable wavelength tunability in both directions and a much improved tunable wavelength range of ~190 nm compared to the ~100 nm tunable range in proposed system with monodirectional control. While DEAs present a limitation requiring high voltages for CLCE wavelength tuning, this issue can potentially be alleviated by (i) enhancing the dielectric permittivity or (ii) lowering the tangent modulus of the DEA material, or iii) optimizing the CLCE by reducing their thickness or lowering the elastic modulus. These approaches are expected to reduce the driving voltage of proposed systems for practical applications. In brief, the findings of this study confirm the long-time desired omnidirectional wavelength control of tunable structural color and stretchable CLCEs, overcoming the research limitation of the previous focus on solely mono-directional wavelength tuning control. By maximizing the tunable functionality and degrees of freedom via omnidirectional wavelength control, we foresee multiple potential innovations in various wavelength-tunable photonic applications, such as displays, multiple-wavelength optical sensors, and photonic camouflage.

## Materials and methods

### CLCE synthesis

Stretchable CLCEs were synthesized using a combination of the thiol-acrylate chain reaction and bar-coating method. The chiral reactive mesogen (LC756, BASF) and diacrylate reactive mesogen (RM257, GRANDINCHEM) were dissolved in solvent (toluene, Sigma-Aldrich) and heated at 85 °C for 5 min. After cooling the solution to room temperature, the thiol chain extender (EDDET, Sigma-Aldrich), thiol crosslinker (PETMP, Sigma-Aldrich), photoinitiator (Irgacure 651, Sigma-Aldrich), and catalyst dipropylamine (DPA, Sigma-Aldrich) were mixed and vigorously vortexed until the appropriate viscosity for bar coating was obtained. The prepared solution was then bar-coated on the conventional OHP film using an applicator with a 220 μm gap at a speed of 11 mm s^-1^. Finally, if the intended reflection color was apparent, photo-polymerization was conducted in an ultraviolet chamber for 5 min at an intensity of 30 mW cm^-2^ and a central wavelength of 365 nm (CSM1010, AUVCURE). The synthesized CLCE was easily delaminated onto the OHP film using tweezers. As a result, free-standing CLCEs with a thickness of 81 μm can be fabricated.

### Preparation of DEA

A commercial dielectric elastomer film (VHB 4910) was pre-stretched using a biaxial stretching jig. To retain the required pre-stretched state, a rigid acryl frame was applied to the pre-stretched elastomer film. Conductive carbon paste was then placed on the top and bottom sides of the elastomer film as compliant electrodes. A compliant electrode was also placed on the surface of the dielectric elastomer film using a mask with an appropriate structure based on the deformation mode of the DEA. The synthesized CLCE was pre-stretched according to the desired areal pre-stretching amount and attached to the top of the elastomer film. Copper tape was employed at the end of the carbon paste on the acrylic frame to enable voltage application. The high-voltage amplifier was placed on a copper strips.

### Electro-optical and electro-mechanical characterization

A high-voltage electrical source was generated using an arbitrary function generator AFG1022 (Tektronix, USA) and voltage amplifier 609B (Tektronix). The generated electrical signal was monitored using an oscilloscope (TBS2000B, Tektronix). Optical microscopy equipment (BX53M, Olympus, Japan), a complementary metal-oxide-semiconductor image sensor camera (HAWK-SCM63, Zootos, USA), and a high-resolution spectrometer (HR4 Pro. Ocean Insight, USA) were used to measure the optical characteristics of the CLCE. A laser displacement sensor (LJ-V7200, Keyence, Japan) was used to measure the actuation characteristics of the DEA (Fig. S6).

### Finite element analysis

We further verified the operation of the contraction-mode DEA using commercial simulation software (COMSOL Multiphysics 5.6) for finite element analysis. The elastic modulus, thickness, and radius of the dielectric elastomer film (VHB 4910) were measured experimentally for precise calculations. By applying a voltage to the compliant electrode of the DEA, the areal contractile deformation was confirmed. Moreover, the calculated distribution of the principal strain was consistent with the experimental results.

### Supplementary information

Supplementary Information

Supplementary Video S1

Supplementary Video S2

Supplementary Video S3

Supplementary Video S4

Supplementary Video S5

Supplementary Video S6
