# Supplementary material for: Omnidirectional color wavelength tuning of stretchable chiral liquid crystal elastomers
Source: Light Sci Appl. 2024 May 22;13:114. doi: 10.1038/s41377-024-01470-w (PMC11109264; doi:10.1038/s41377-024-01470-w)
Supplement: Supplementary file 1 — Supplementary Information [file 41377_2024_1470_MOESM1_ESM.docx]

**Supplementary information**

**Omnidirectional Color Wavelength Tuning of Stretchable Chiral Liquid Crystal Elastomers**

**Supplementary Information for**

**Omnidirectional Color Wavelength Tuning of Stretchable Chiral Liquid Crystal Elastomers**

*Seungmin Nam, Wontae Jung, Jun Hyuk Shin, and Su Seok Choi^*^*

Department of Electrical Engineering, Pohang University of Science and Technology (POSTECH), Pohang, 37673, Korea

Corresponding author: [choiss@postech.ac.kr](mailto:choiss@postech.ac.kr) (Su Seok Choi)


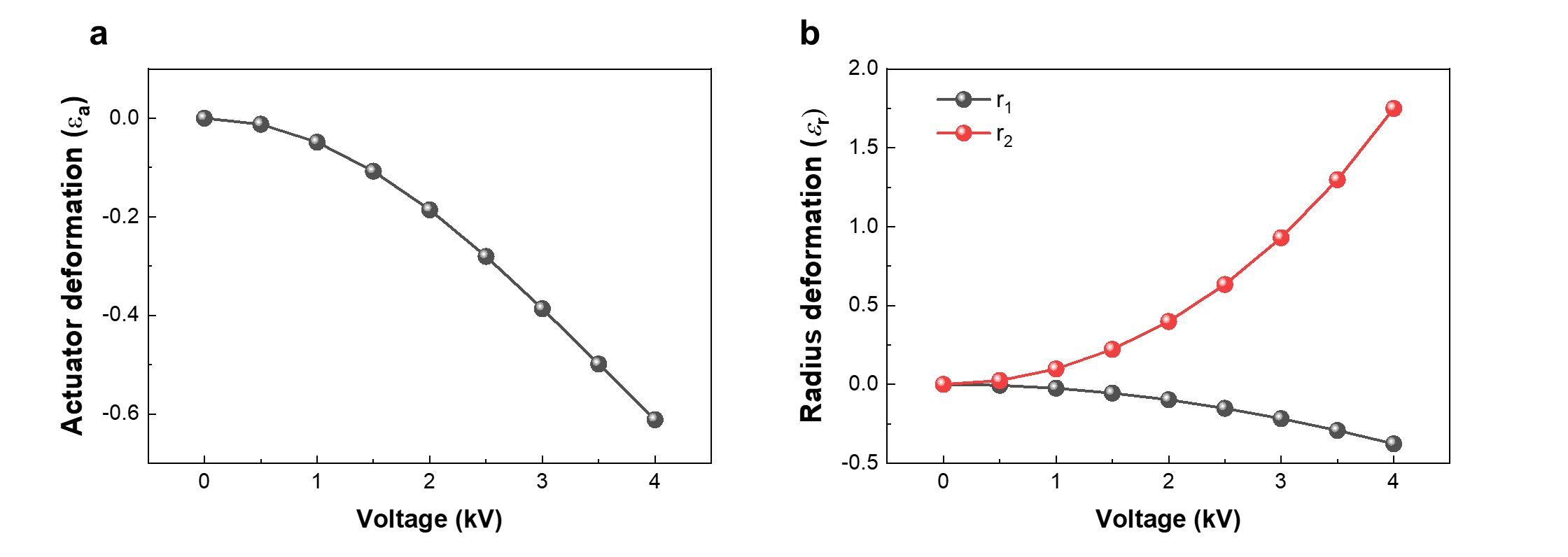


**Figure S1.** FEA simulation results for contraction mode DEA operation. (a) areal strain, and (b) radial strain amount as a function of applied voltage. As a result of inner circle of *r*_1_ decreasing deformation, contraction deformation followed. Note that outer radius increased whereas the inner radius decreased. (See Figures 2b and 2g)


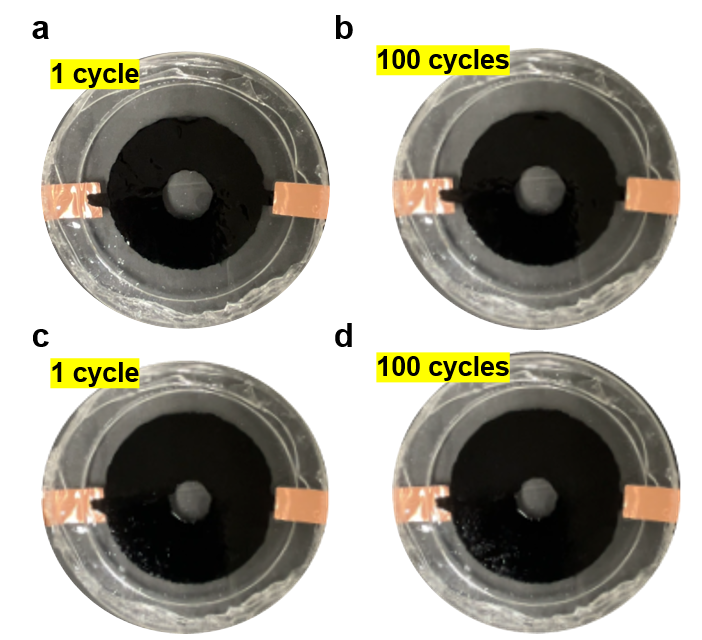


**Figure S2.** Repeatability test of compressive donut-shape DEA actuator results up to 100 cycle operation. a-d) Photographs of contraction mode DEA at 1 cycle, no deformation (a), 100 cycles, no deformation (b), 1 cycle, maximum deformation (c), and 100 cycles, maximum deformation (d).


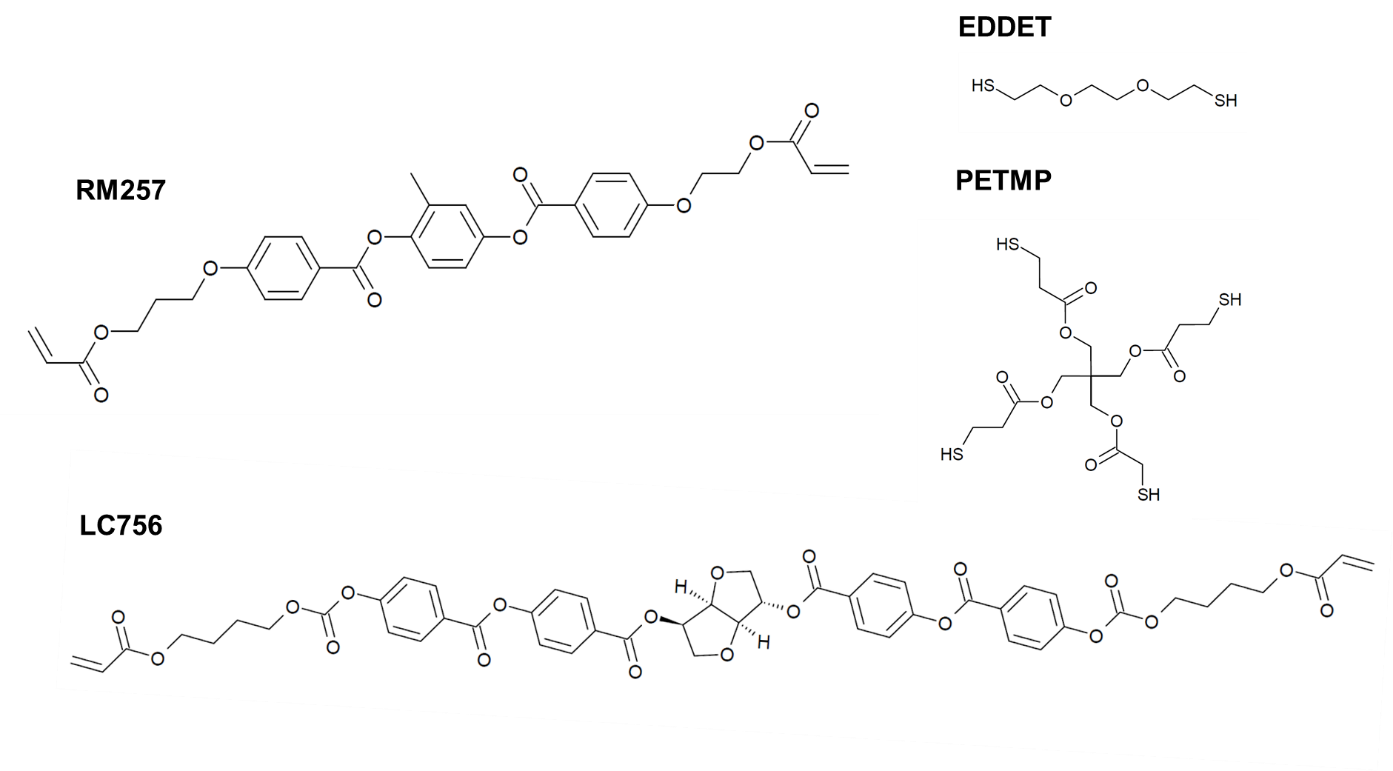


**Figure S3.** Chemical structure of materials used to synthesize CLCE precursor.

**
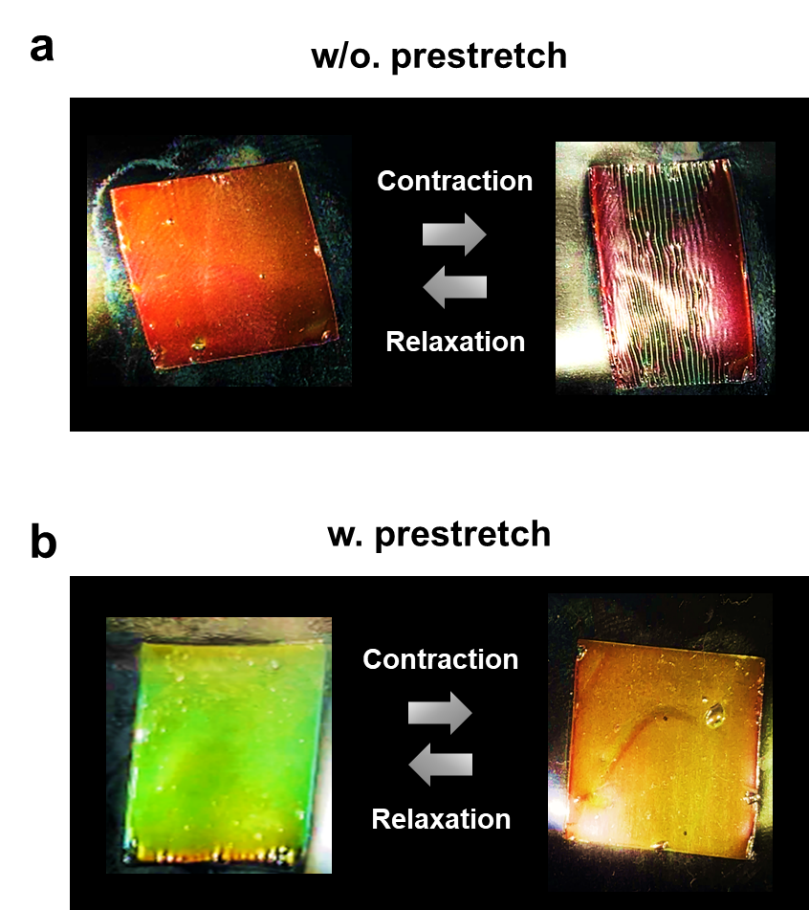
**

**Figure S4.** Difference in color change when contraction deformation was applied with and without prestretching of CLCE. a) When pre-stretch was not applied to CLCE, only small amount of color tuning can be observed and side effect of out-of-plane buckling was observed on the CLCE surface. b) When pre-stretch was sufficiently applied to CLCE, full and continuous color tuning was confirmed.

**
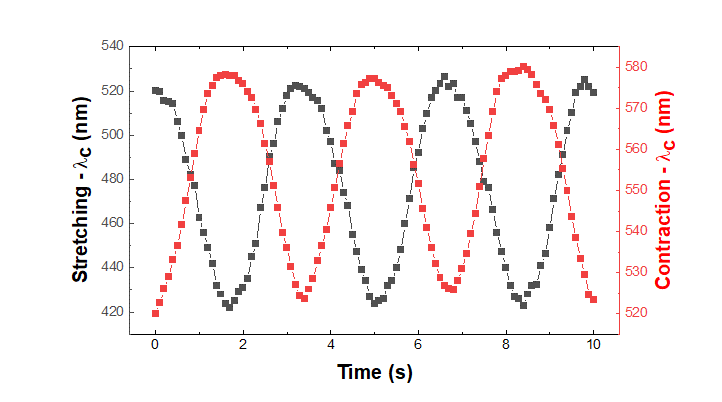
**

**Figure S5.** Time-based monitoring of central wavelength changes behaviors of omnidirectional mode CLCE during 10 seconds with sine wave voltage of 3.7 kV.

**
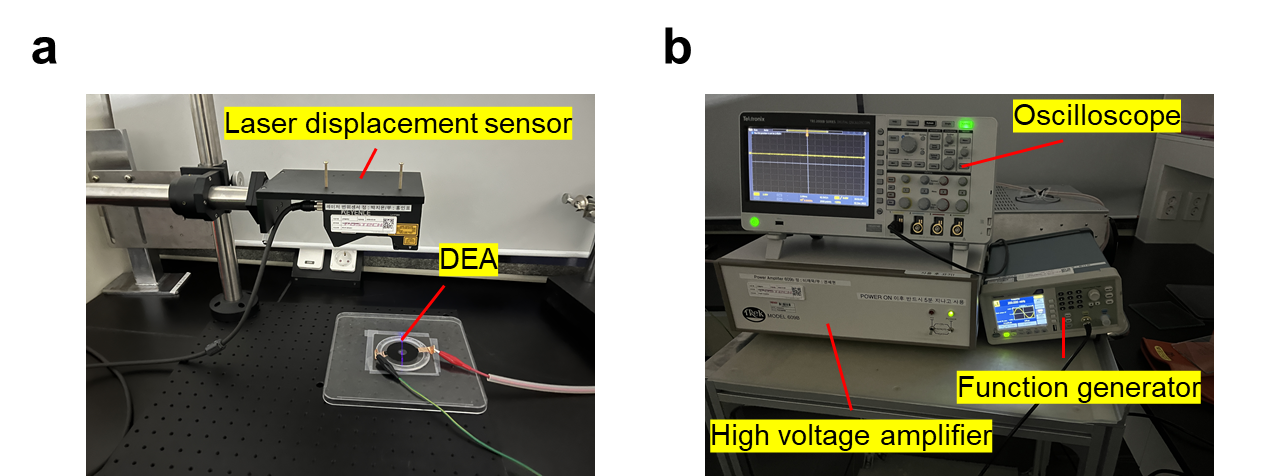
**

**Figure S6.** Experimental setup for electro-mechanical characterization and high-voltage amplification. a) Laser displacement sensor setup for actuation performance measurement. b) Lab-made high-voltage amplifier setup.

*
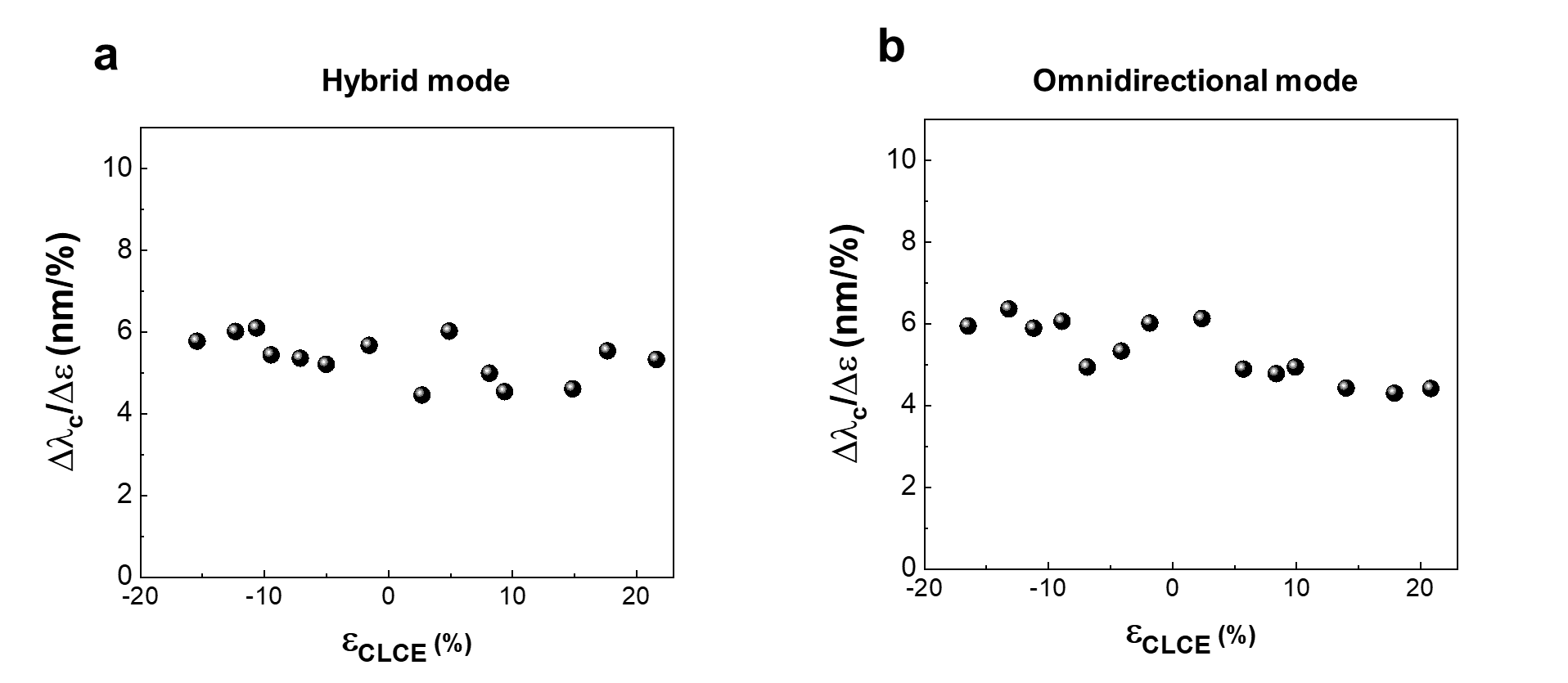
* **Figure S7.** Color-strain sensitivity of (a) hybrid in Figure 5 (b) and omnidirectional mode CLCE in Figure 6.


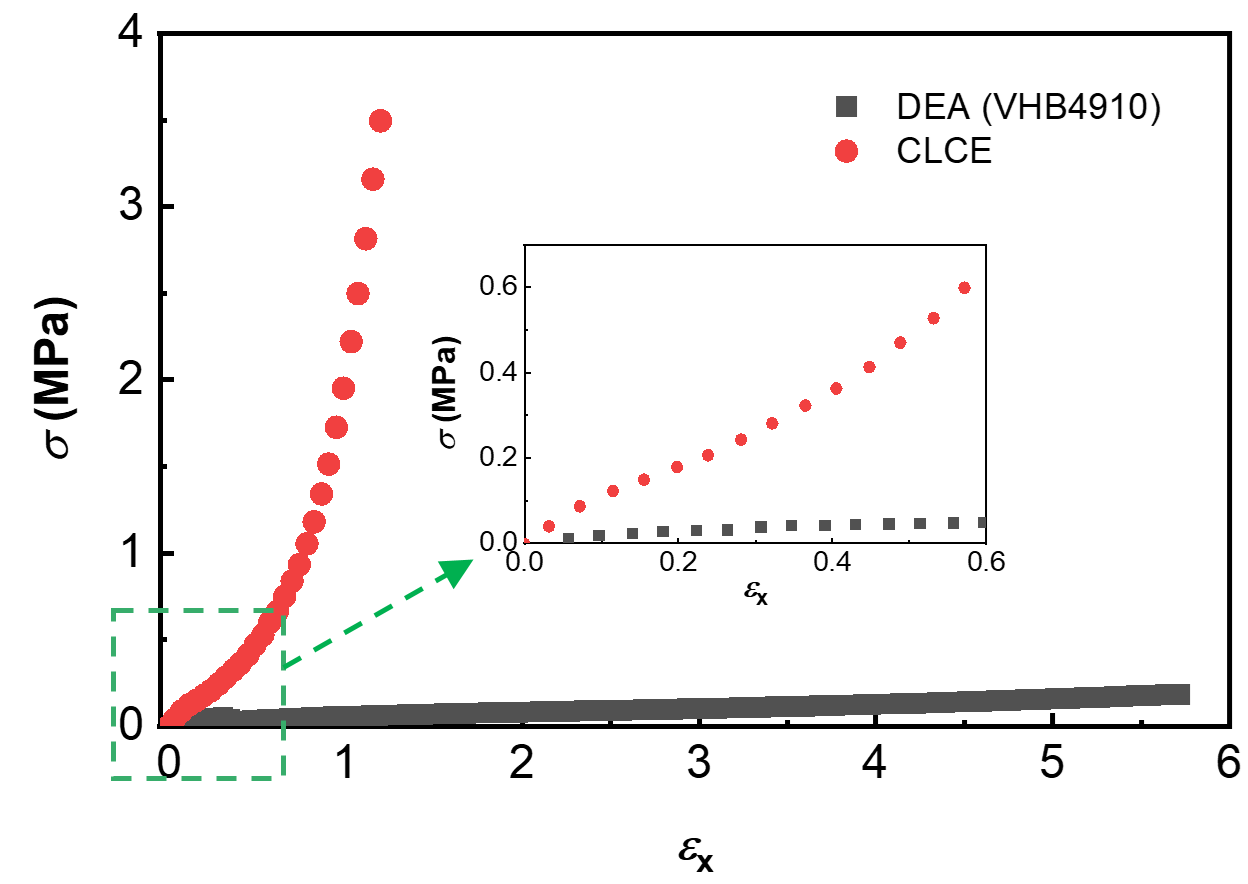


**Figure S8.** Stress-strain curve of the DEA and CLCE under uniaxial tensile strain.

*
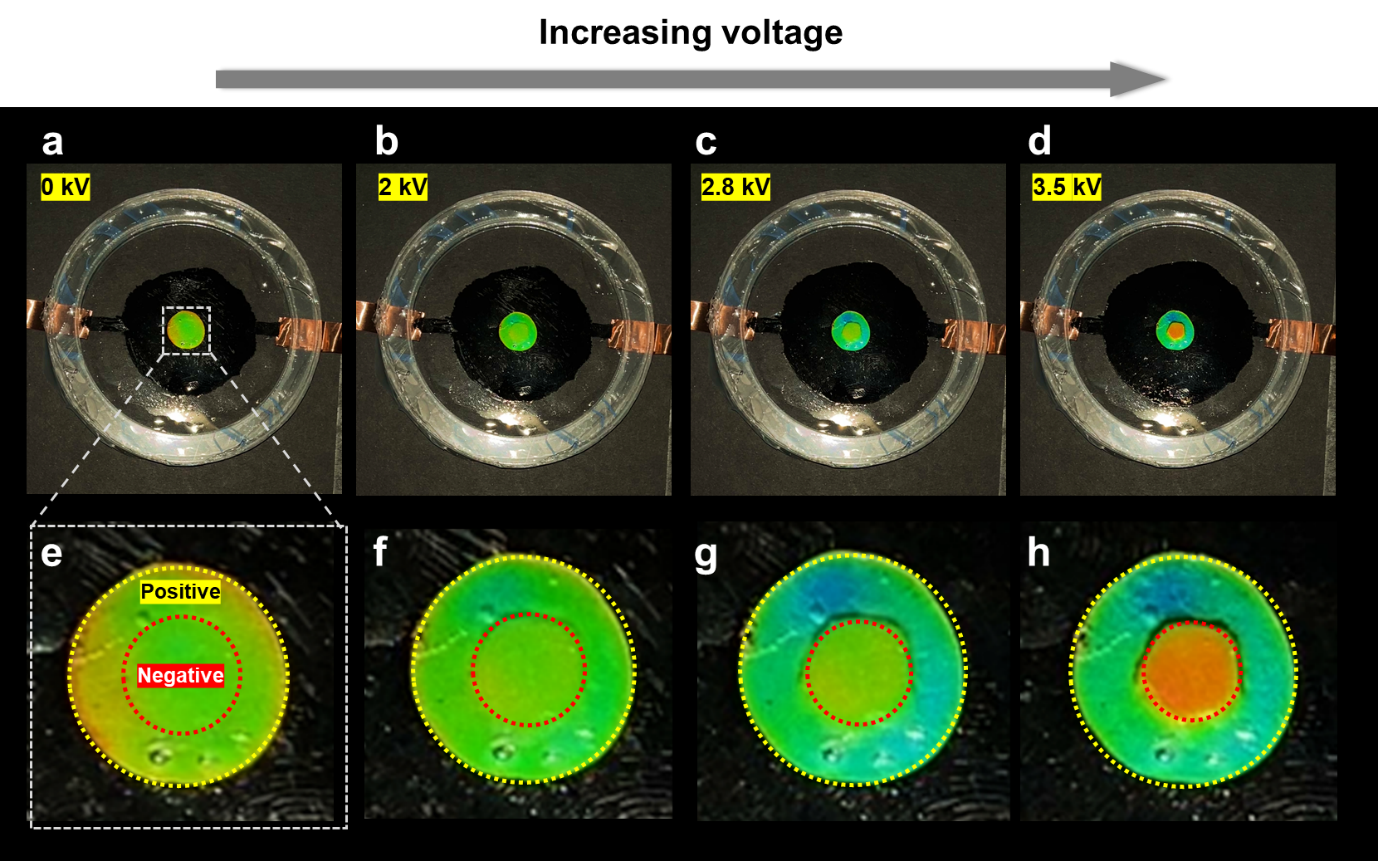
*

**Figure S9.** Structural color changes when a single CLCE is subjected to both negative and positive strains simultaneously. Images of the device at various applied voltages of 0, 2, 2.8, and 3.5 kV, respectively (a-d). Close-up images of the CLCE at various applied voltages of 0, 2, 2.8, and 3.5 kV, respectively (e-h).

**Supplementary Video 1 |** **Operation of contraction mode DEA (Simulation)**

**Supplementary Video 2 | Operation of contraction mode DEA (Experiment)**

**Supplementary Video 3 |** **Structural color tuning of CLCE toward long-wavelength direction**

**Supplementary Video 4 |** **Structural color tuning of hybrid mode CLCE**

**Supplementary Video 5 |** **Real time spectrum shifting of omnidirectional mode CLCE**

**Supplementary Video 6 |** **Structural color tuning of omnidirectional mode CLCE**
